# Tribenzotriquinacene Fused Dimers (“Fusodimers”)—Synthesis and Host–Guest Properties

**DOI:** 10.1002/chem.71574

**Published:** 2026-08-17

**Authors:** Chun‐Fai Ng, Lisi He, Yin‐Ying Cheung, Xiao‐Ping Cao, Dietmar Kuck, Hak‐Fun Chow

**Affiliations:** ^1^ Department of Chemistry The Chinese University of Hong Kong Shatin New Territories Hong Kong; ^2^ State Key Laboratory of Applied Organic Chemistry and College of Chemistry & Chemical Engineering, Lanzhou University Lanzhou P.R. China; ^3^ Department of Chemistry and Center for Molecular Materials (CM_2_) Bielefeld University Bielefeld Germany

**Keywords:** fullerene complexes, molecular bowls, polyaromatic host compounds, supramolecular chemistry, tribenzotriquinacenes

## Abstract

The synthesis and structural properties of two stereoisomeric tribenzotriquinacene (TBTQ) derivatives, *syn*‐**4** and *anti*‐**4**, in which two rigid TBTQ bowls share a common benzene ring, are described. Owing to the orthogonal orientation of the three indane wings in each of the TBTQ units, four veratrole rings point either at right angles or parallel into the 3‐space. In contrast to the convex‐concave *anti*‐isomer, the biconcave fusodimer *syn*‐**4** was found to host C_60_ and C_70_ fullerenes as 1:1 complexes both in solution and in the solid state, as shown by ^1^H NMR spectroscopy and x‐ray crystallography. Complexation‐induced chemical shift (CIS) measurements gave similar binding constants, *K*_a_ = 382 (± 4) for *syn*‐**4**⊂C_60_ and *K*_a_ = 410 (± 8) M^−^^1^ for *syn*‐**4**⊂C_70_. Structural details of the torsion and widening of the formally *C*_2v_‐symmetric host skeleton upon embedding of the fullerenes into the deep biconcave molecular surface of *syn*‐**4** were determined along with intermolecular peculiarities of the solid‐state complexes.

## Graphical abstract

Two tribenzotriquinacene units merged with each other in either *syn*‐ or *anti*‐orientation of the bowls have become accessible in a short synthesis. In contrast to the *anti*‐isomer, the *syn*‐isomer forms 1:1 host–guest complexes with both C_60_ and C_70_ in solution and in the solid state, as shown by ^1^H NMR spectroscopy and x‐ray diffraction. The Cartesian orientation of the four electron‐rich wings is maintained in both “fusodimers”.

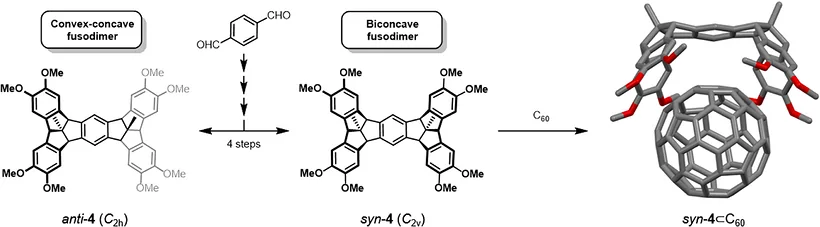

## 1 Introduction

Tribenzotriquinacene **1** (TBTQ, Figure 1), a structurally unique molecule bearing three indane units oriented orthogonally to each other in space [1, 2, 3, 4, 5], has been used as a versatile building block towards the construction of many molecular bowls [6, 7, 8, 9, 10, 11, 12, 13] and capsules [14, 15, 16, 17, 18, 19, 20, 21]. As the TBTQ framework possesses only a relatively small molecular cavity, the molecule itself is not an effective host for molecular binding. However, binding strength and profile can be enhanced by the addition of various appendages to the indane wings [6, 7, 8, 11]. For example, triptycene‐functionalized TBTQ **2** [6] and tris(thianthreno)triquinacene **3** [7, 11] were found to form 1:1 and/or 1:2 complexes with C_60_ and/or C_70_, with binding constants (*K*_1_ or *K*_2_) in the range of 3 × 10^2^ to 6 × 10^3^ M^−1^ [22]. Even the extension of the TBTQ core with three TBTQ‐based centrohexaindane units has been achieved with the resulting complex with C_60_ having a *K*_1_ = 1.5 × 10^4^ M^−1^ [23].

**FIGURE 1:**
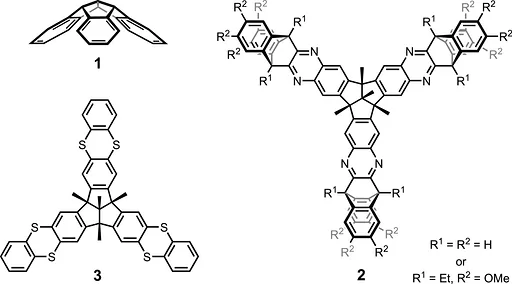
Structures of parent TBTQ **1** (side view) and wing‐extended TBTQ hosts **2** and **3**.

We envisaged that stitching two or several TBTQ bowls into a single, yet larger molecular entity could be an attractive strategy towards the construction of effective hosts. While the non‐covalent or reversibly covalent assembly approaches have been shown to produce aesthetically pleasing TBTQ‐based molecular capsules [14, 15, 16, 17, 18, 19, 20, 21], the resulting molecular ensembles mostly do not possess robust stability to withstand changes in the molecular environment such as temperature, solvent polarity, and pH values, and their guest binding and capsulation behavior are yet to be unfolded. To our knowledge, there is only one report of a self‐assembled TBTQ‐octamer that acted as a host for tetraalkylammonium ions and the pyrene molecule [17].

## 2 Results and Discussion

Herein we report the short synthesis of two diastereomeric tribenzotriquinacenes that bear two mutually covalently linked TBTQ units, *syn*‐**4** and *anti*‐**4**, from readily available starting materials in four steps, based on the established sequence involving 2‐benzyhydryl‐substituted 1,3‐indanediols (Figure 2) [1, 5, 24]. Actually, these two compounds are not truly dimeric but represent truncated dimers, which we coin “fusodimers” here, with the TBTQ cores sharing one aromatic ring. In fact, the same molecular skeleton, but with different and unlike substitution patterns in the two molecular moieties of *syn*‐**5** and *anti*‐**5**, had already been reported by Hopf and coworkers using a different and relatively long synthesis strategy [10]. In their report, apart from the x‐ray crystal data of isomer *anti*‐**5**, no additional information with regard to the host guest properties of these dimers were disclosed. Here we provide further structural and host–guest complexation properties of both of the newly prepared fusodimers *syn*‐**4** and *anti*‐**4**. As it turns out, due to the larger molecular cavity and the deeper concave shape, isomer *syn*‐**4** is a highly effective host for fullerenes such as C_60_ and C_70_. Hence, compounds **4** can serve as alternative building blocks, similar to the parent TBTQ **1** and its derivatives, to be used in the construction of complex molecular systems, and further π‐extended warped polyaromatic compounds [25, 26, 27].

**FIGURE 2:**
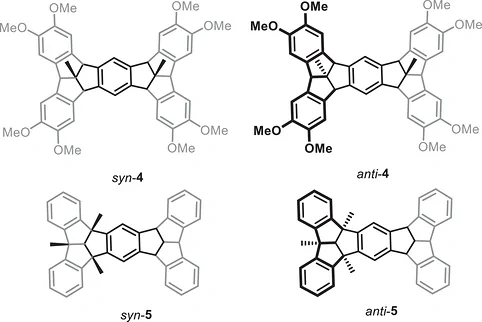
Structures of the diastereomeric pairs of TBTQ fusodimers **4** and **5**.

The synthesis of the fusodimers **4** began with 4‐bromoveratrole **6**, which was treated with *n*‐butyllithium, followed by two‐fold reaction with terephthalaldehyde **7** to give diol **8** in 78% yield as a mixture of stereoisomers (Scheme 1). The mixture was subjected to double condensation with indanedione **9** [28, 29] in the presence of *para*‐toluenesulfonic acid to obtain the tetraketones **10** in 72% yield. Compounds **10** were reduced with lithium aluminum hydride to give the tetraalcohols **11** in 99% yield. Whereas the product **10** was isolated as a mixture of diastereomers, intermediate **11** was a particularly complex mixture and used without purification. The final step of the synthesis was the four‐fold cyclodehydration of tetrol **11** using orthophosphoric acid in xylenes as a solvent. Stereoisomer s*yn*‐**4** was obtained in 13% yield by repeated recrystallization of the product mixture from methanol, while isomer *anti*‐**4** was isolated from the combined mother liquors by column chromatography in 10% yield. The summed product yield of **4** (23%) obtained with the bis‐indanediols **11** represents a remarkably good outcome in view of the yields of comparable two‐fold cyclodehydration reactions achieved with simple and similarly electron‐rich 2‐benzhydyryl‐1,3‐indanediols (45%−50%) [28, 29].

**SCHEME 1:**
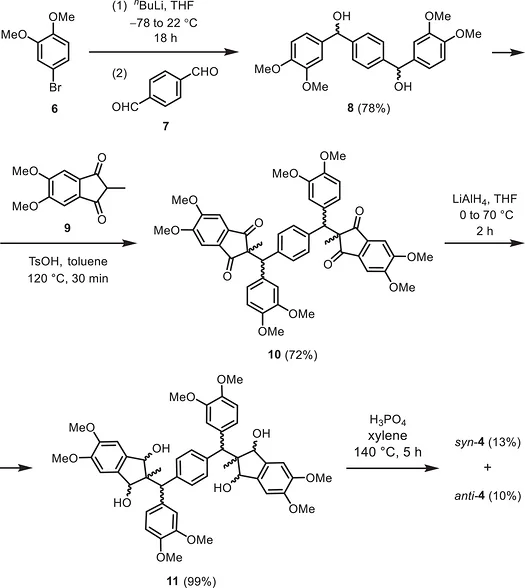
Synthesis of the fusodimers *syn*‐**4** and *anti*‐**4**.

The structural identities and spectral assignments of all compounds were confirmed by combined ^1^H and ^13^C NMR spectroscopy and mass spectrometry. As expected, the intermediates **8**, **10** and **11** were mixtures of diastereomers and full NMR signal assignment was not possible, but the relative chemical shift values within specific regions and the relative integrals matched well with the expected structures. The mass spectrometric data obtained by electrospray ionization (ESI) confirmed the presence of the molecular ions [M + Na]^+^ of all new compounds with the expected *m*/*z* values (see Supporting Information).

The structures of the title compounds *syn*‐**4** and *anti*‐**4** were ascertained by ^1^H and ^13^C NMR and 2D NMR spectroscopy, mass spectrometry and x‐ray crystallography (see Figures S1–S4 and Table S1). For the *syn*‐**4** dimer, the ^1^H NMR spectrum shows the presence of three sets of isolated arene protons through the singlets at δ 7.44, 6.89, and 6.79 ppm. Also, two singlets at δ 4.33 and 4.25 ppm reflect the two sets of bridgehead protons, two singlets at δ 3.81 and 3.80 ppm indicate the two different sets of methoxy groups, and a singlet at δ 1.67 ppm is due to the methyl protons (Figure 3, top). For the *anti*‐**4** dimer, the respective groups of protons give rise to slightly different chemical shifts (Figure 3, bottom). While the methoxy resonances of *syn*‐**4** are almost isochronous (Δ*δ* = 0.01 ppm), those of *anti*‐**4** are separated slightly further (Δ*δ* = 0.06 ppm). Notably, the reverse relation holds true for the two sets of bridgehead protons (Δ*δ* = 0.09 vs. 0.92 ppm). In both cases, the ^1^H NMR spectra nicely confirm the molecular symmetries of the isomers (*syn*‐**4**: *C*_2v_, *anti*‐**4**: *C*_2h_).

**FIGURE 3:**
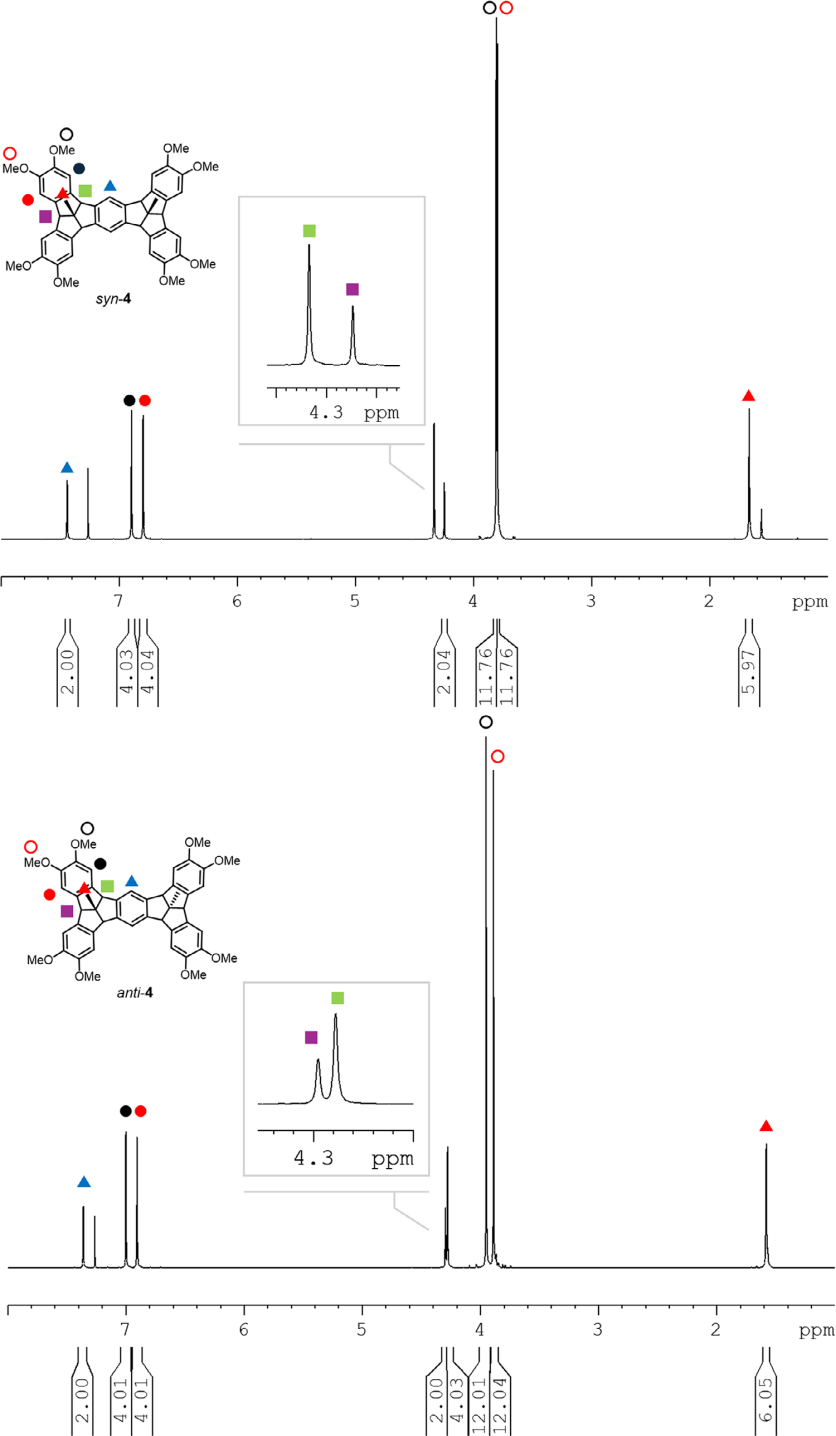
^1^H NMR spectra (500 MHz, CDCl_3_) of the TBTQ fusodimers *syn*‐**4** and *anti*‐**4**.

Slow evaporation of a toluene solution of *syn*‐**4** afforded single crystals suitable for x‐ray diffraction analysis, which revealed the presence of a solvate bearing one molecule of *syn*‐**4** and one of toluene in the asymmetric unit. The molecular structure consists of two TBTQ units that share a benzene ring (**A**) within the central *s*‐indacene unit with the apical methyl groups oriented toward the same hemisphere (Figure 4a) [30]. The width **XY** and the depth of the concavity of *syn*‐**4** were measured as 7.09 and 3.73 Å, respectively. The ideal *C*_2v_ symmetry of the polycyclic framework is reduced to *C*_1_ as the molecule is slightly twisted. The angle (∠_ind‐ind_) between two indane planes shared within the indacene unit was measured as 6.9°. The angle (∠**XAY**) between two axes of the inner indane moieties was found to be 177.7°, indicating a slight outward bending of the *s*‐indacene unit. The angles between the axes of the three indane units of one TBTQ moiety, **XA**, **XB**, and **XC**, and those between the corresponding axes **YA**, **YD**, and **YE** of the other one ranged from 84.4° to 89.8°, thus deviating slightly from perfect orthogonality. Moreover, the dihedral angle between two apical methyl groups ∠_Me‐Me_ was determined as 6.0°, also differing to some extent from the ideal value of 0° (Figure 4b). A molecule of toluene was found to be encapsulated in the cavity with the closest distance of 3.45 Å. In the crystal packing, molecules of *syn*‐**4** and the translation equivalents form a linear chain along the *b*‐axis through C−H···O interactions with a length of 2.65 Å (Figure 4c). Moreover, the glide reflection equivalent of each *syn*‐**4** molecule formed another linear chain along the *b*‐axis so that C−H··· π interactions with distances in the range of 2.91 to 2.93 Å were witnessed between neighboring chains.

**FIGURE 4:**
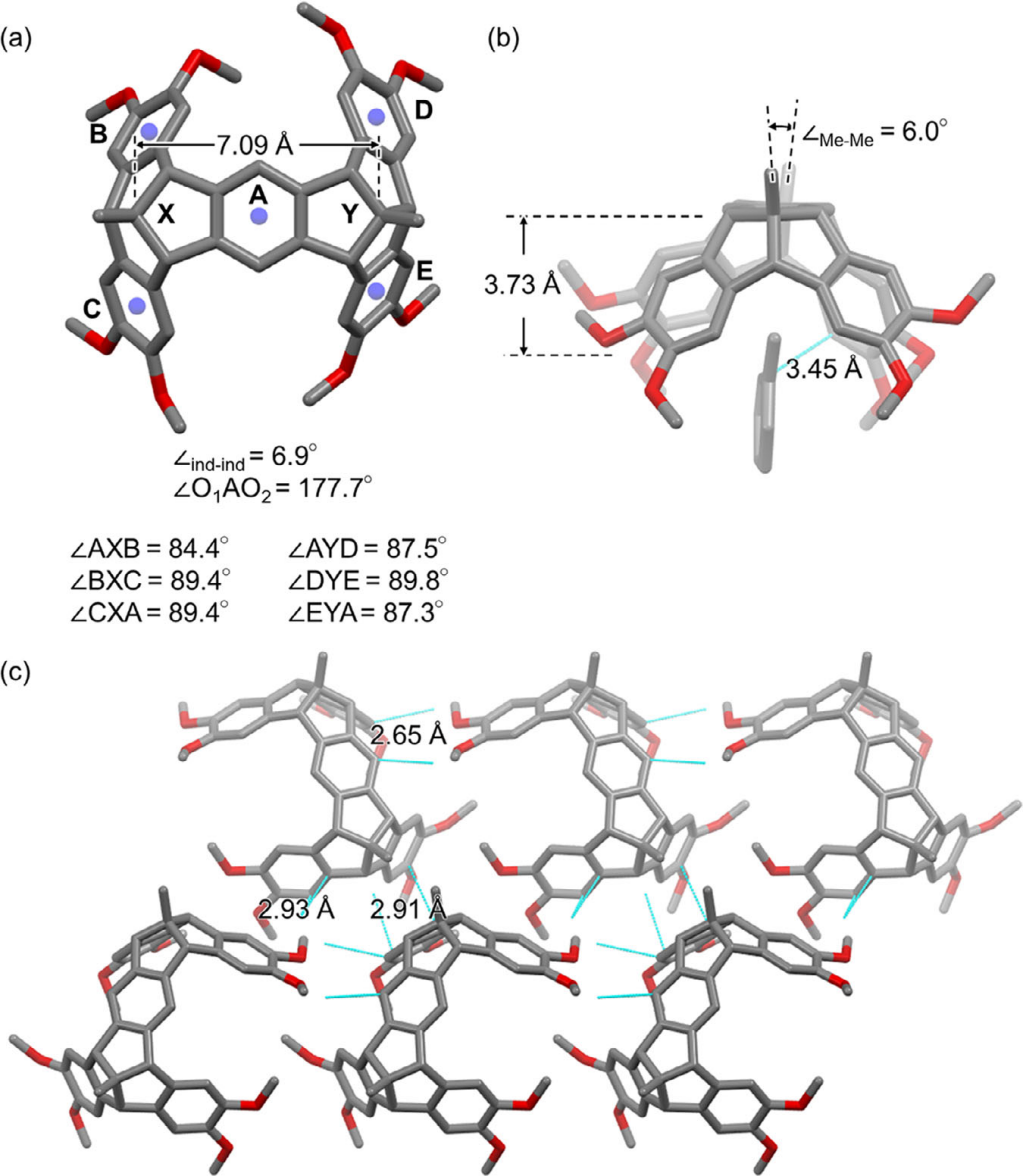
X‐ray crystal structure of TBTQ fusodimer *syn*‐**4**: (a) Top view showing the width the cavity and the angles between the axes of the indane units, the centroids of the benzene rings are indicated in blue; (b) side view showing the depth of the cavity, the twisting of concave skeleton and the encapsulation of a molecule of toluene; (c) linear chain observed in the crystal packing. Hydrogen atoms are omitted for clarity.

Single crystals of *anti*‐**4** suitable for x‐ray structural analysis were obtained from 1,1,2,2‐tetrachloroethane (TCE) solution. X‐ray diffraction analysis revealed that there is only half of the *anti*‐**4** molecule together with four TCE solvates in the asymmetric unit [30]. Since the centroid of the *anti*‐**4** molecule sits on the inversion center, two separated conical cavities by TBTQ cores are inverted to each other with the expected *C*_2h_ symmetry. The vertical separation between the concave up‐oriented TBTQ core and the concave down‐oriented counterpart was measured as 5.65 Å. The width and the depth of two identical conical cavities are 8.27 and 2.96 Å, respectively (Figure 5a). The angles between the three axes of indane units **XA**, **XB**, and **XC** and their symmetrical equivalents (**xa**, **xb**, and **xc**) range from 87.9° to 92.3°, indicating slightly less deviation from perfect orthogonality and inward bending within the TBTQ cores as compared to *syn*‐**4**. Unlike *syn*‐**4**, no molecular twisting was observed as the dihedral angle (∠_Me‐Me_) was found to be 180°. Moreover, the two central indane planes are coplanar, as the angles (∠_ind‐ind_) and (∠**XAx**) are 0° and 180°, respectively, because of the inversion symmetry (Figure 5b). Short contacts between the veratrole rings (**X**···**x**’ and **Y**···**y**’) of two adjacent *anti*‐**4** molecules allow the formation of infinite chains. The stacking is not parallel, and the intermolecular distance of the two closest rings was measured to be 3.49 Å. Two solvated TCE molecules are accommodated in each of the conical cavities of a given *anti*‐**4** molecule with intermolecular distances of 3.43 Å (Figure 5c). Another two TCE molecules are coordinated to the two apical methyl groups along the chain with a distance of 3.49 Å.

**FIGURE 5:**
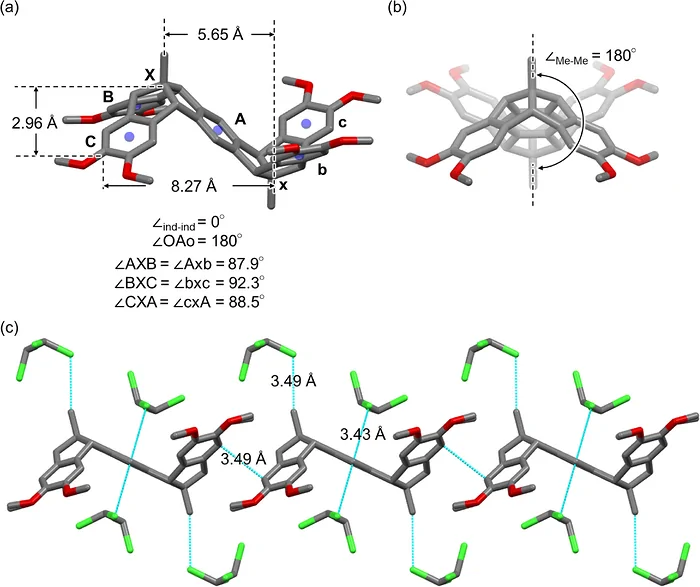
X‐ray crystal structure of TBTQ fusodimer *anti*‐**4**: (a) Side view showing the width and the depth of the two conical cavities and the angles between the axes of the indane units, the centroids of the benzene rings are indicated in blue; (b) side view showing the lack of twisting of the skeleton; (c) linear chain with the different accommodation of TCE solvate molecules observed in the crystal packing. All the solvates and hydrogen atoms are omitted for clarity.

We expected that, from the sizes of the cavity shown in the x‐ray crystal structures of *syn*‐**4** and *anti*‐**4**, these two compounds may be capable of binding fullerenes. Job plot and/or complexation‐induced chemical shift (CIS) measurements were performed to determine the binding stoichiometry and binding constants in the solution state using toluene‐*d*_8_ as the solvent. The Job plots indicated a 1:1 binding stoichiometry in both cases involving *syn*‐**4** with C_60_ and C_70_ (Figures S6 and S10). The most pronounced downfield shifts of ^1^H NMR resonances were noted for the two sets of equivalent methoxy protons, and the concentration‐dependent chemical shift values were fitted into a 1:1 binding model [31, 32], giving binding constants *K*_a_ of 382 (± 1.1%) and 410 M^−^^1^ (± 2.0%) for C_60_ and C_70_, respectively (Figures S5, S7, S9, and S11). This indicates that fusodimer *syn*‐**4** has similar binding affinity towards the fullerene guests C_60_ and C_70_. On the other hand, no observable trend could be obtained from CIS spectroscopy involving *anti*‐**4** with either C_60_ or C_70_ **_,_** suggesting that this stereoisomer is not an effective host for fullerenes (Figure S8). In comparison to the more extended TBTQ‐fullerene complexes mentioned in the Introduction, fusodimer *syn*‐**4** exhibits relatively weak binding due to the less pronounced cavity of its polycyclic framework.

Crystals suitable for x‐ray structural analysis were also obtained from toluene solutions of the host–guest complexes of *syn*‐**4**⊂C_60_ and *syn*‐**4**⊂C_70_ [30]. X‐ray diffraction analysis of *syn*‐**4**⊂C_60_ revealed that the twisting of the skeleton of *syn*‐**4** is reduced upon encapsulation of a C_60_ molecule, as the dihedral angle ∠_Me‐Me_ decreases from 6.0° to 4.2° (Figure 6a). After complexation with C_60_, the angle (∠_ind‐ind_) increases significantly from 6.9° to 16.6° while the angle (∠**XAY**) decreases from 177.7° to 168.1°, which is likely due to the steric congestion of the host by the bulky fullerene guest (Figure 6b). A slight outward bending of the *s*‐indacene unit was observed as the width of the cavity decreased to 7.02 Å. The angles between the axes of the indane units, **XA**, **XB**, and **XC**, as well as **YA**, **YD**, and **YE** ranged from 88.0° to 91.7°, suggesting that almost ideal orthogonality of the TBTQ cores is attained upon complexation with C_60_. The distances between the centroid of C_60_ and the central benzene ring **A** of *syn*‐**4** were determined as 7.08 Å, and the corresponding distances to the benzene rings **B**, **C**, **D**, and **E** of the other four TBTQ units were found to be 7.08, 6.67, 6.50, 6.68, and 6.67 Å, respectively. Offset π‐π stackings were observed where the distances between the periphery of C_60_ and the four dimethoxyindane wings range within 3.17−3.18 Å. Moreover, π–π stacking between C_60_ and wing **B’** of a neighboring molecule of *syn*‐**4** was also observed with a distance of 6.74 Å (Figure 6c). Interestingly, pairs of two sideward adjacent C_60_ guest molecules exhibit short contacts with centroid‐to‐centroid distances of 9.94 Å. However, only dimeric subunits but not infinite chains of C_60_ are formed, as both ends of such pairs were solvated by toluene molecules at the periphery of C_60_.

**FIGURE 6:**
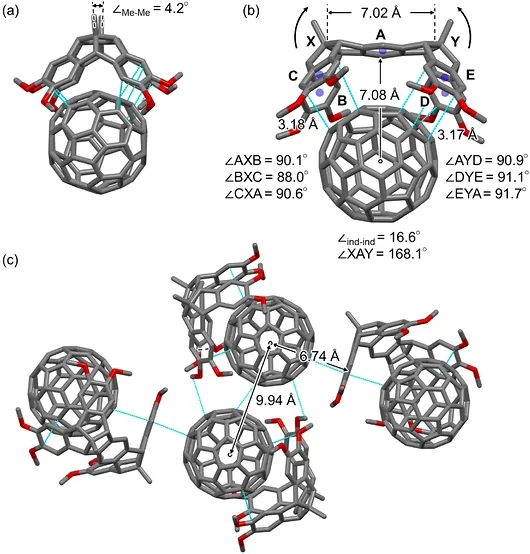
X‐ray crystal structure of host–guest complex *syn*‐**4**⊂C_60_: (a) Side view showing the slight twisting of the concave skeleton; (b) side view showing the width and depth of the cavity and the angles between the axes of indane units upon the encapsulation of C_60_; the centroids of the benzene rings are shown in blue; (c) dimeric subunits observed in the crystal packing. All solvate molecules and hydrogen atoms are omitted for clarity.

Encapsulation of a C_70_ molecule instead of C_60_ was found to reduce the twisting of the molecular skeleton of host *syn*‐**4** even further. X‐ray diffraction analysis of *syn*‐**4**⊂C_70_ revealed that the dihedral angle (∠_Me‐Me_) is only 2.1° in this case (Figure 7a). Outward bending of two central indane planes was also observed, with the angles (∠_ind‐ind_ and ∠**XAY**) measured as 13.9° and 170.0°, respectively, while the width **XY** of the cavity remain at 7.04 Å (Figure 7b). Similar to the complexation of *syn*‐**4** with C_60_, the angles between three axes of indane units **XA**, **XB**, and **XC** as well as **YA**, **YD**, and **YE** range from 86.4° to 91.6°, again suggesting almost ideal orthogonality within each of the TBTQ cores upon complexation of *syn*‐**4** with C_70_. A subtle difference in the encapsulation mode of C_70_ with host *syn*‐**4** was found in comparison with that of C_60_. Intuitively, the orientation of the ellipsoidal C_70_ guest within the complex should be co‐axial with the host *syn*‐**4**, such that the major axis of the fullerene would be oriented perpendicular to the central benzene ring **A**. However, a tilt with an angle (∠_tilt_) of 15.5° was observed between the major axis of the ellipsoid of C_70_ and the perpendicular of central benzene ring **A** of *syn*‐**4**. The distances between the centroid of C_70_ and the five benzene rings **A** and **B**−**E** of host *syn*‐**4** were measured as 7.61, 6.94, 7.04, 6.83, and 6.72 Å, respectively. Offset π‐π stackings were also observed in the crystal of *syn*‐**4**⊂C_70_, where the distances between the periphery of C_70_ and the four dimethoxyindane wings range from 3.04 to 3.39 Å. Furthermore, π‒π stacking between C_70_ of a given complex *syn*‐**4**⊂C_70_ and the benzene ring **B’** of the host of a neighboring complex was observed with a distance of 7.25 Å, slightly longer as compared to the case of *syn*‐**4**⊂C_60_ (Figure 7c). In further analogy, dimeric subunits were also observed in the crystal packing of *syn*‐**4**⊂C_70_. Two neighboring C_70_ molecules exhibit short contacts with centroid‐to‐centroid distances of 10.0 Å.

**FIGURE 7:**
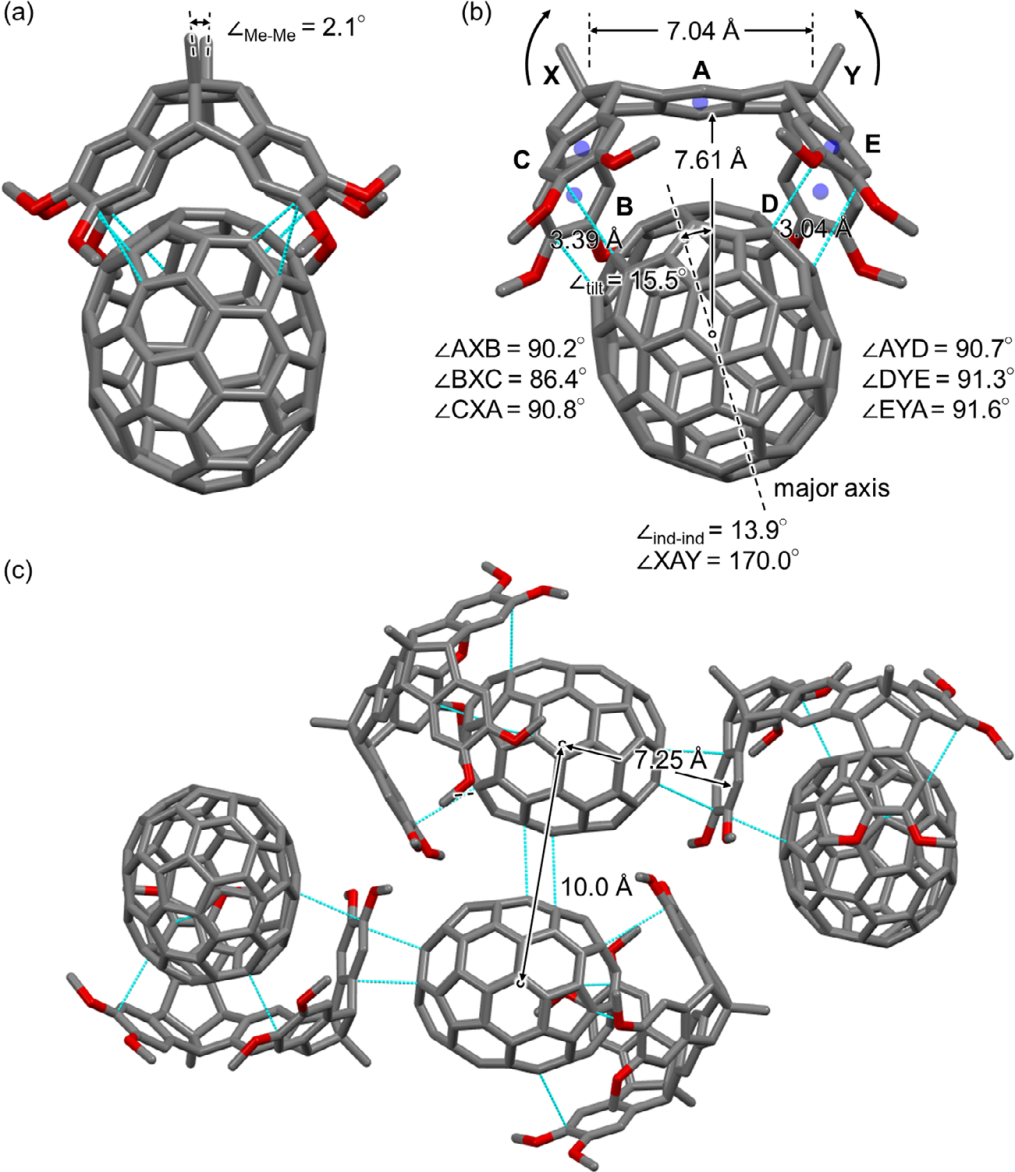
X‐ray crystal structure of host–guest complex *syn*‐**4**⊂C_70_: (a) Side view showing the slight twisting of the concave skeleton; (b) side view showing the width and depth of the cavity and the angles between the axes of the indane units upon the encapsulation of C_70_; (c) dimeric subunit observed in the crystal packing. All solvate molecules and hydrogen atoms are omitted for clarity.

## 3 Conclusion

In summary, we have disclosed an efficient, short synthesis of two symmetrically functionalized tribenzotriquinacene dimers, *syn*‐**4** and *anti*‐**4**, involving four‐fold cyclodehydration in the last step. The two concave TBTQ moieties share a single central benzene ring, with the two cavities of these “fusodimers” being oriented either to the same or to two different hemispheres. Both title compounds were fully characterized by NMR spectroscopy, mass spectrometry, and x‐ray crystallography. The large biconcave framework of *syn*‐**4**, containing five electron‐rich arene rings, provides a large polyaromatic pocket and is well suited for the encapsulation of the fullerene molecules C_60_ and C_70_ with similar binding strength. In contrast, stereoisomer *anti*‐**4** was found to be devoid of any binding capabilities with fullerenes, obviously due to the unfavorable mutual orientation of the two TBTQ bowls. The association constants and structural details of the 1 : 1 host–guest complexes *syn*‐**4**⊂C_60_ were elucidated by CIS spectroscopy and x‐ray crystallography, respectively. The presence of the eight methoxy groups at the peripheral veratrole entities in both *syn*‐**4** and *anti*‐**4** can provide handles for further refunctionalization and extension of the rigid polycyclic framework. Even the six unsubstituted benzhydrylic bridgeheads of these particular fusodimers may be utilized for further modulation at later stages. Owing to the intrinsic orthogonality of the TBTQ core, the perpetuated Cartesian geometry of fused TBTQ oligomers may be used for the development of novel unusual 3D‐nonplanar polyaromatic compounds.

## Conflicts of Interest

The authors declare no conflicts of interest.

## Supporting information



Additional supporting information can be found online in the Supporting Information section. The authors have cited an additional reference within the Supporting Information [33].
**Supporting File 1**: chem71574‐sup‐0001‐SuppMat.docx

## Data Availability

The data that support the findings of this study are available on request from the corresponding author. The data are not publicly available due to privacy or ethical restrictions.
