# Supplementary material for: Tribenzotriquinacene Fused Dimers (“Fusodimers”)—Synthesis and Host–Guest Properties
Source: Chemistry. 2026 Aug 17;32(41):e71574. doi: 10.1002/chem.71574 (PMC13730004; doi:10.1002/chem.71574)
Supplement: Supplementary file 1 [file CHEM-32-e71574-s001.docx]

Tribenzotriquinacene Fused Dimers (“Fusodimers”) – Synthesis and Host-Guest Properties

Chun-Fai Ng, ^[a]^ Lisi He, ^[a]^ Yin-Ying Cheung, ^[a]^ Xiao-Ping Cao,*^[b]^, Dietmar Kuck*^[c]^ and Hak-Fun Chow*^[a]^

^[a]^ Department of Chemistry, The Chinese University of Hong Kong, Shatin, NT, Hong Kong

^[b]^ State Key Laboratory of Applied Organic Chemistry and College of Chemistry & Chemical Engineering, Lanzhou University, Lanzhou 730000, P. R. China

^[c]^ Department of Chemistry and Center for Molecular Materials (CM_2_), Bielefeld University, 33615 Bielefeld, Germany

**Table of Contents**

1. General …………………………………………………………………………………... S2

2. Syntheses and characterization data of the new compounds …………………………...... S3

3. 2D ^1^H–^1^H NOESY spectra of compounds *syn*-**4** and *anti*-**4**……….……………….…… S7

4. X-ray crystallography ..........................................…………………………………….... S11

5. Study of host-guest binding by ^1^H NMR spectroscopy ……………………………….... S12

6. NMR and mass spectra of the new compounds ………………………………............... S17

7. CheckCIF of crystals: *syn*-**4**, *anti*-**4**, *syn*-**4**⊂C_60_ and *syn*-**4**⊂C_70_………………………... S30

8. References …………………………………………………………..………………….. S42

**1. General**

All reagents were purchased from commercial suppliers and used without further purification. Tetrahydrofuran (THF) and toluene were freshly distilled from sodium/benzophenone ketyl and sodium, respectively, under nitrogen before use. Unless otherwise noted, all reactions were carried out under nitrogen protection gas. All reactions were monitored by thin layer chromatographic (TLC) analysis on EM Science silica gel 60 F254 aluminum plates, which were visualized by a UV lamp at 254 or 365 nm and/or stained using dodecamolybdophosphoric acid in ethanol followed by heating. Flash column chromatography was performed on glass column of silica gel (230–400 mesh). NMR spectra were recorded on a Bruker Advance III 500 spectrometer (^1^H: 500.13 MHz; ^13^C: 125.76 MHz). Chemical shifts were reported as parts per million on the *δ* scale using the solvent (residual) resonance as internal standard. Data are represented as follows: Chemical shift, integration, multiplicity (s = singlet, d = doublet, t = triplet, q = quartet, m = multiplet, br = broad), and coupling constant (*J*, Hz). High resolution mass spectra (HRMS) were obtained on a Bruker SolariX 9.4T ICR mass spectrometer using electrospray ionization (ESI). UV-vis spectra were recorded on a Shimadzu UV-3600 Plus spectrophotometer. Melting points were measured on a digital melting point apparatus and are uncorrected.

**2. Syntheses and characterization data for new compounds**

**5,6-Dimethoxy-2-methyl-1*H*-indene-1,3(2*H*)-dione (Compound 9):**^[1, 2]^ Eaton’s reagent (60 mL, 9.1 w% of P_4_O_10_ in methanesulfonic acid) was added to a mixture of veratrole (6.90 g, 50 mmol) and methylmalonic acid (7.10 g, 60 mmol) in CH_2_Cl_2_ (120 mL). The resulting mixture was stirred at 22 °C for 18 h. It was then was poured into ice-water (250 mL) and the products were extracted with CH_2_Cl_2_ (4 × 300 mL). The organic phase was concentrated under reduced pressure and the crude product was washed with EtOH to afford the target compound **9** (7.25 g, 33 mmol, 66%) as a pale yellow solid. The spectral data are in accordance with those reported in the literature.^[1,2]^

**1,4-Phenylenebis((3,4-dimethoxyphenyl)methanol) (Compound 8):** *n*-Butyllithium (1.6 M in hexane, 30.0 mL, 48 mmol) was added dropwise to the solution of 4-bromoveratrole (11.3 g, 52 mmol) in dry THF (250 mL) at –78 °C. The resulting mixture was stirred at –78 °C for 30 min, and terephthalaldehyde (2.68 g, 20 mmol) was added in solid form in one portion. The cooling bath was then removed, and the mixture was stirred at 22 °C for 18 h under nitrogen. The reaction was quenched by cautiously adding saturated aqueous NH_4_Cl (200 mL) and the resulting mixture was stirred at 22 °C for 30 min before extraction with diethyl ether (3 × 200 mL). The combined organic layers were washed with water (100 mL), dried over anhydrous Na_2_SO_4_, filtered, concentrated under reduced pressure. The crude pale-yellow residue obtained was washed with hexane (2 × 100 mL) and then with CH_2_Cl_2_/hexane (1:1) (3 × 50 mL) to afford compound **8** (5.10 g, 12.4 mmol, 62%) as an off-white solid. The washed filtrates were combined, concentrated under reduced pressure and the residue was purified by flash column chromatography (hexane/EtOAc = 1/1 to 1/3) to afford a further crop of compound **8** (1.30 g, 3.2 mmol, 16%) as an off-white solid. Hence, the total yield of compound **8** was 78%, m.p.: 174–175 °C; *R*_f_: 0.30 (hexane/EtOAc =1/3); ^1^H NMR (500 MHz, CDCl_3_): δ = 7.33 (s, 4 H, ArH), 6.91 (d, *J* = 1.9 Hz, 2 H, ArH), 6.86 (dd, *J* = 8.2, 1.9 Hz, 2 H, ArH), 6.80 (d, *J* = 8.2 Hz, 2 H, ArH), 5.77 (d, *J* = 3.4 Hz, 2 H, CH), 3.85 (s, 6 H, OCH_3_), 3.83 (s, 6 H, OCH_3_), 2.30 (d, *J* = 3.4 Hz, 2 H, OH) ppm; ^13^C NMR (126 MHz, CDCl_3_): δ = 149.2, 148.6, 143.3, 143.2, 136.5, 126.7, 126.7, 119.0, 119.0, 111.0, 109.8, 75.9, 56.0, 56.0 ppm; HRMS (ESI): *m*/*z* calcd. for C_24_H_26_O_6_Na^+^ ([M + Na]^+^): 433.1622, found: 433.1625 (Δ = + 0.6 ppm).

**2,2'-(1,4-Phenylenebis((3,4-dimethoxyphenyl)methylene))bis(5,6-dimethoxy-2-methyl-1*H*-indene-1,3(2*H*)-dione) (****Compound 10, mixture of stereoisomers):** A solution of indane-1,3-dione **9** (4.85 g, 22 mmol), compound **8** (4.1 g, 10 mmol) and *p*-toluenesulfonic acid monohydrate (0.38 g, 2 mmol) in toluene (200 mL) was heated to reflux for 30 min. The mixture was then allowed to cool. The solvent was removed under reduced pressure and the residue was re-dissolved in CH_2_Cl_2_ (500 mL). The resulting solution was then washed with saturated aqueous Na_2_CO_3_ (4 × 200 mL) and water (100 mL), dried over anhydrous Na_2_SO_4_, filtered, and concentrated under reduced pressure. The residue was purified by flash column chromatography (hexane/EtOAc/CH_2_Cl_2_ = 1/1/1 to EtOAc/CH_2_Cl_2_ = 1/1) to afford compound **10** (5.87 g, 7.2 mmol, 72%) as a pale-yellow solid, m.p.: 261–262 °C; *R*_f_: 0.32 (hexane/EtOAc/CH_2_Cl_2_ = 1/1/1); ^1^H NMR (500 MHz, CDCl_3_): δ = 7.25 (s, 2 H, ArH), 7.24 (s, 2 H, ArH), 7.11 (s, 1 H, ArH), 7.11 (s, 1 H, ArH), 7.10 (s, 1 H, ArH), 7.09 (s, 1 H, ArH), 7.04 (s, 1 H, ArH), 7.02 (s, 1 H, ArH), 6.77 (d, *J* = 8.1 Hz, 2 H, ArH), 6.59 (d, *J* = 8.3 Hz, 2 H, ArH), 4.34 (s, 2 H, CH), 3.93 (s, 3 H, OCH_3_), 3.92 (s, 3 H, OCH_3_), 3.91 (s, 3 H, OCH_3_), 3.88 (s, 3 H, OCH_3_), 3.79 (s, 3 H, OCH_3_), 3.79 (s, 3 H, OCH_3_), 3.74 (s, 3 H, OCH_3_), 3.74 (s, 3 H, OCH_3_), 1.09 (s, 3 H, CH_3_), 1.08 (s, 3 H, CH_3_) ppm; ^13^C NMR (126 MHz, CDCl_3_): δ = 203.4, 203.4, 203.3, 203.2, 155.9, 155.9, 155.9, 155.9, 148.3, 147.6, 147.6, 138.6, 138.6, 136.6, 136.6, 136.6, 136.5, 132.6, 132.5, 129.5, 129.4, 122.0, 121.9, 112.9, 112.9, 110.8, 103.1, 103.1, 103.0, 102.9, 58.0, 58.0, 57.0, 57.0, 56.7, 56.6, 55.8, 55.7, 19.7, 19.6 ppm; HRMS (ESI): *m*/*z* calcd. for C_48_H_46_O_12_Na^+^ ([M + Na]^+^): 837.2881, found: 837.2883 (Δ = + 0.2 ppm).

**2,2'-(1,4-Phenylenebis((3,4-dimethoxyphenyl)methylene))bis(5,6-dimethoxy-2-methyl-2,3-dihydro-1*H*-indene-1,3-diol) (Compound 11, mixture of stereoisomers),:** Lithium aluminium hydride (2.10 g, 54 mmol) was added cautiously to a solution of compound **10** (9.78 g, 12 mmol) in anhydrous THF (100 mL) at 0 °C. The cooling bath was removed, and the reaction mixture was heated to reflux for 2 h. Then the mixture was cooled to 0 °C and diluted with diethyl ether (200 mL). Water (5 mL) and aqueous NaOH (1 M, 5 mL) were added dropwise sequentially at 0 °C. The mixture was then allowed to warm to 22 °C and stirred for 1 h. Then the mixture was dried over anhydrous Na_2_SO_4_, filtered through a short pad of silica gel under rinsing with diethyl ether, concentrated under reduced pressure and dried *in vacuo* to afford the compound **11** as a pale-brown foamy solid (9.80 g, 11.9 mmol, 99%). The product was used directly in the subsequent step without further purification. M.p.: 147–148 °C; *R*_f_: 0.42 (EtOAc); HRMS (ESI): *m*/*z* calcd. for C_48_H_54_O_12_Na^+^ ([M + Na]^+^): 845.3507, found: 845.3513 (Δ = + 0.7 ppm). Based on to the absence of an expectable peak at *m*/*z* 843, it is obvious that all the carbonyl groups were reduced.

**TBTQ fusodimers *syn*-4 and *anti*-4**: A mixture of orthophosphoric acid (85%, 30 mL) and xylene (100 mL) was heated to reflux for 30 min in a flask equipped with a dropping funnel. A solution of the crude tetraalcohol **11** obtained in the previous step **11** (8.23 g, 10 mmol) in xylene (100 mL), formed by gentle warming, was then added dropwise over 1 h with stirring. After the addition was complete, the mixture was stirred and heated to reflux for 4 h. The mixture was allowed to cool and the xylene solution was decanted carefully into a round bottom flask. Xylene (50 mL) was added to the remaining phosphoric acid mixture, and the mixture was stirred under gentle warming with a heat gun, then the xylene layer was decanted, and this process was repeated twice. The combined xylene solutions were concentrated under reduced pressure affording a brown solid, which was re-dissolved in MeOH (50 mL) under reflux. The solution was then cooled in an ice bath and filtered, and the solid collected was washed with cold MeOH. This process was repeated twice to afford *syn***-4** (976 mg, 1.30 mmol, 13%) as an off-white solid. The filtrates were combined and concentrated under reduced pressure and the residue was purified by flash column chromatography (hexane/EtOAc = 1/1 to hexane/EtOAc/CH_2_Cl_2_ = 1/1/1) to afford *anti***-4** (751 mg, 1.00 mmol, 10%) as an off-white solid.

**(4b^1^*rs,*9b^1^*sr*)-2,3,6,7,11,12,15,16-Octamethoxy-4b^1^,9b^1^-dimethyl-4b,4b^1^,8b,9b,9b^1^,13b,17b,18b-octahydrodibenzo[2,3:4,5]pentaleno[1,6-*ab*]dibenzo[2,3:4,5]pentaleno[1,6-*gh*]-*s*-indacene (TBTQ fusodimer *syn*-4):** M.p.: 300 °C (dec.); *R*_f_: 0.27 (hexane/EtOAc/CH_2_Cl_2_ = 2/1/1); ^1^H NMR (500 MHz, CDCl_3_): δ = 7.44 (s, 2 H, ArH), 6.89 (s, 4 H, ArH), 6.79 (s, 4 H, ArH), 4.33 (s, 4 H, CH), 4.25 (s, 2 H, CH), 3.81 (s, 12 H, OCH_3_), 3.80 (s, 12 H, OCH_3_), 1.67 (s, 6 H, CH_3_) ppm; ^13^C NMR (126 MHz, CDCl_3_): δ = 149.6, 149.3, 145.2, 137.7, 137.2, 120.1, 108.2, 107.6, 63.5, 63.1, 63.0, 56.7, 56.4, 27.7 ppm; HRMS (ESI): *m*/*z* calcd. for C_48_H_46_O_8_Na^+^ ([M + Na]^+^): 773.3085, found: 773.3087 (Δ = + 0.2 ppm).

**(4b^1^*rs,*9b^1^*rs*)-2,3,6,7,11,12,15,16-Octamethoxy-4b^1^,9b^1^-dimethyl-4b,4b^1^,8b,9b,9b^1^,13b,17b,18b-octahydrodibenzo[2,3:4,5]pentaleno[1,6-*ab*]dibenzo[2,3:4,5]pentaleno[1,6-*gh*]-*s*-indacene (TBTQ fusodimer *anti*-4)::** M.p.: 375 °C (dec.); *R*_f_: 0.56 (hexane/EtOAc/CH_2_Cl_2_ = 2/1/1); ^1^H NMR (500 MHz, CDCl_3_): δ = 7.36 (s, 2 H, ArH), 7.00 (s, 4 H, ArH), 6.90 (s, 4 H, ArH), 4.30 (s, 2 H, CH), 4.28 (s, 4 H, CH), 3.95 (s, 12 H, OCH_3_), 3.89 (s, 12 H, OCH_3_), 1.58 (s, 6 H, CH_3_) ppm; ^13^C NMR (126 MHz, CDCl_3_): δ = 149.6, 149.4, 145.4, 138.0, 137.2, 120.1, 108.2, 107.5, 63.4, 63.1, 63.0, 56.9, 56.4, 27.6 ppm; HRMS (ESI): *m*/*z* calcd. for C_48_H_46_O_8_Na^+^ ([M + Na]^+^): 773.3085, found: 773.3083 (Δ = − 0.2 ppm).

**3. 2D ^1^H**–**^1^H NOESY** **spectra of compounds *syn*-4 and *anti*-4**

**Figure S1.** 2D ^1^H–^1^H NOESY spectrum (500 MHz, CDCl_3_, 298 K) of *syn*-**4**.

**Figure S2.** Partial 2D ^1^H–^1^H NOESY spectra (500 MHz, CDCl_3_, 298 K) of *syn*-**4**.

**Figure S3.** 2D ^1^H–^1^H NOESY spectrum (500 MHz, CDCl_3_, 298 K) of *anti*-**4**.

**Figure S4.** Partial 2D ^1^H–^1^H NOESY spectra (500 MHz, CDCl_3_, 298 K) of *anti*-**4**.

**4. X-ray crystallography**

Crystallographic data of *syn*-**4**, *anti*-**4**, *syn*-**4**⊂C_60_ and *syn*-**4**⊂C_70_ were collected on a Bruker D8 VENTURE diffractometer with Mo Kα radiation (λ = 0.71073 Å) from a sealed tube generator. Data collection, reduction, and empirical absorption corrections were performed using APEX4 software. The crystal structure was solved by direct methods with the SHELXS program,^[3]^ and all non-hydrogen atoms were anisotropically refined against *F^2^* with full-matrix least-squares techniques using the SHELXL-97 program. All the hydrogen atoms were included in the structure factor calculation at idealized positions with fixed isotropic thermal displacement parameter relative to the attached atoms.

**Table S1. Crystallographic data**

| Compound | *anti*-**4** | *syn*-**4** | *syn*-**4**⊂C_60_ | *syn*-**4**⊂C_70_ |
| --- | --- | --- | --- | --- |
| Formula | C_48_H_46_O_8_∙8(C_2_H_2_Cl_4_) | C_48_H_46_O_8_∙C_7_H_8_ | 2(C_48_H_46_O_8_) ⊂2C_60_∙7(C_7_H_8_) | 2(C_48_H_46_O_8_) ⊂2C_70_∙7(C_7_H_8_) |
| Formula weight | 2093.53 | 842.98 | 3587.83 | 3828.03 |
| Crystal system | Orthorhombic | Monoclinic | Monoclinic | Monoclinic |
| Space group | *Pbca* (No. 61) | *C*2/c (No. 15) | *P*2_1_/*n* (No. 14) | *P*2_1_/*n* (No. 14) |
| Temperature / K | 173(2) | 100(2) | 100(2) | 100(2) |
| *a* / Å | 13.3589(4) | 40.277(10) | 13.596(4) | 13.6482(3) |
| *b* / Å | 20.6501(7) | 11.3227(15) | 24.813(9) | 26.1482(7) |
| *c* / Å | 31.5341(11) | 22.300(3) | 24.638(6) | 24.6276(7) |
| *α / °* | 90 | 90 | 90 | 90 |
| *β* / ° | 90 | 118.899(8) | 90.216(6) | 90.8844(8) |
| *γ* / ° | 90 | 90 | 90 | 90 |
| *V /* Å^3^ | 8699.1(5) | 8903(3) | 8312(4) | 8788.0(4) |
| *Z* | 4 | 8 | 2 | 2 |
| *D*_c_ (g/cm^3^) | 1.599 | 1.258 | 1.433 | 1.447 |
| *F*(000) | 4216 | 3584 | 3732 | 3972 |
| *R*_int_ | 0.0228 | 0.0283 | 0.0650 | 0.0797 |
| GOF | 1.042 | 1.026 | 1.051 | 1.072 |
| Measured reflections | 57797 | 72306 | 98368 | 134858 |
| Independent reflections | 7855 | 8049 | 15022 | 15903 |
| *R*_1_ (*I* > 2*σ*(*I*))*^a^* | 0.0312 | 0.0401 | 0.0531 | 0.0485 |
| *R*_1_ (all data) | 0.0377 | 0.0462 | 0.0780 | 0.0798 |
| *wR*_2_ (*I* > 2*σ*(*I*))*^b^* | 0.0774 | 0.1011 | 0.1211 | 0.1074 |
| CCDC number | 2427155 | 2165284 | 2165286 | 2165287 |

^a^*R*_1_ = Σ||*F*_o_| − |*F*_c_|| / Σ|*F*_o_|

^b^*wR*_2_ = [Σ[*w*(*F*_o_^2^ − *F*_c_^2^ )^2^] / Σ*w*(*F*_o_^2^ )^2^]^1/2^

**5. Study of host-guest binding by ^1^H NMR spectroscopy**

For the ^1^H NMR titration experiment with C_60_, 500 μL of the solution of host *syn*-**4** (1.0 mM in toluene‑*d*_8_) was added to an NMR tube. This solution was titrated against a 30.0 mM solution of the guest C_60_ containing 1.0 mM of the host **4**. An equilibration time of around 3 min was allowed after each addition of the titrant before the ^1^H NMR spectrum was recorded. The solvent residual signal at δ 2.08 was used as the internal standard. The experimental data were analysed using the bindfit program available from the Internet website http://supramolecular.org to obtain the association constant *K*_a_.

**Figure S5.** Stacked ^1^H NMR spectra (500 MHz, toluene-*d*_8_, 298 K, 1.0 mM) of *syn*-**4** recorded during the titration with C_60_.

**Figure S6.** Job Plot for the binding of host *syn*-**4** with guest C_60_ (toluene-*d*_8_, 298 K, total concentration 1.0 mM), revealing 1 : 1 binding stoichiometry.

**Figure S7.** Global fitting of the experimental titration data of *syn*-**4** against C_60_ with the bindfit program available from the Internet website <https://supramolecular.org>.

**Figure S8.** Stacked ^1^H NMR spectra (500 MHz, toluene-*d*_8_, 298 K, 1.0 mM) of *anti*-**4** recorded during the titration with C_60_.

For the ^1^H NMR titration experiment with C_70_: 500 μL of the solution of host *syn-***4** (0.5 mM in toluene-*d*_8_) was added to an NMR tube. This solution was titrated against a 30.0 mM solution of the guest C_60_ containing 0.5 mM of the host *syn*-**4**. An equilibration time of around 3 min was allowed after each addition of the titrant before the ^1^H NMR spectrum was recorded. The solvent residual signal at δ 2.08 was used as the internal standard. The experimental data were analysed using the bindfit program available from the Internet website http://supramolecular.org to obtain the association constant *K*_a_.

**Figure S9.** Stacked ^1^H NMR spectra (500 MHz, Toluene-*d*_8_, 298 K, 0.5 mM) of *syn*-**4** recorded during the titration with C_70_.

**Figure S10.** Job Plot for the binding of host *syn*-**4** with guest C_70_ (toluene-*d*_8_, 298 K, total concentration 0.5 mM), showing the 1 : 1 binding stoichiometry.

**Figure S11.** Global fitting of the experimental titration data of *syn*-**4** against C_70_ with the bindfit program available from the Internet website <https://supramolecular.org>.

**5. NMR and ESI mass spectra of new compounds**

Note: The peak group at *m*/*z* 845 indicates overlapping of the signals of ions [M + Na]^+^ and dimeric ions [2 M + 2 Na]^2+^.

Note: The peak group at *m*/*z* 845 indicates overlapping of the signals of ions [M + Na]^+^ and dimeric ions [2 M + 2 Na]^2+^.

Note: The peak group at *m*/*z* 845 indicates overlapping of the signals of ions [M + Na]^+^ and dimeric ions [2 M + 2 Na]^2+^.

**6. CheckCIF of crystals: *syn*-4, *anti*-4, *syn*-4⊂C_60_ and *syn*-4⊂C_70_**

**6. References**

[1] M. Harig, B. Neumann, H.-G. Stammler, D. Kuck, “2,3,6,7,10,11‐Hexamethoxytribenzotriquinacene: Synthesis, Solid‐State Structure, and Functionalization of a Rigid Analogue of Cyclotriveratrylene” *European Journal of Organic Chemistry* **2004** (2004): 2381–2397.

[2] J. Vile, M. Carta, C. G. Bezzu, N. B. McKeown, “Tribenzotriquinacene-based polymers of intrinsic microporosity” *Polymer Chemistry* **2** (2011): 2257–2260.

[3] G. M. Sheldrick, “A short history of SHELX” *Acta Crystallographica. Section A, Foundations of Crystallography* **64** (2007): 112–122.
